# Using Mendelian randomization to investigate a possible causal relationship between adiposity and increased bone mineral density at different skeletal sites in children

**DOI:** 10.1093/ije/dyw079

**Published:** 2016-05-22

**Authors:** John P Kemp, Adrian Sayers, George Davey Smith, Jonathan H Tobias, David M Evans

**Affiliations:** 1 University of Queensland Diamantina Institute, Translational Research Institute, Brisbane, QLD, Australia; 2 MRC Integrative Epidemiology Unit and; 3 School of Clinical Sciences, University of Bristol, Bristol, UK

**Keywords:** Bone, body mass index, genetics, Mendelian randomization, ALSPAC

## Abstract

**Background:** Lean mass is positively associated with bone mineral density (BMD). However, the relationship between adiposity and BMD is more controversial. In particular, it is unclear if the observational association between the two reflects a causal effect of fat mass on BMD. Previous Mendelian randomization (MR) studies using variants in the *FTO* and *MC4R* genes as genetic instruments for adiposity have suggested that fat mass does indeed causally influence BMD. However, it is possible that these genetic variants pleiotropically influence lean mass and affect BMD through pathways independent of adiposity, invalidating one of the core assumptions of MR and complicating interpretation of the analysis.

**Methods:** To investigate whether adiposity causally affects BMD, we investigated the relationship between fat mass and BMD at the skull (SK), upper limbs (UL) and lower limbs (LL), spine (SP) and pelvis (PE), using 32 body mass index (BMI)-associated SNPs, including a variant near *ADCY3* that was strongly associated with fat but not lean mass in our sample. Dual-energy X-ray absorptiometry (DXA) scans and genetic data were available for 5221 subjects (mean age 9.9 years) from the Avon Longitudinal Study of Parents and Children. We performed a series of MR analyses involving single BMI-associated SNPs and allelic scores of these SNPs. We used new extensions of the MR method including MR Egger regression and multivariable MR, which are more robust to possible confounding effects due to horizontal pleiotropy and, in the case of multivariable MR, specifically account for the effect of lean mass in the analysis. Bidirectional Mendelian randomization analysis was also performed to examine whether BMD causally affected BMI and adiposity.

**Results:** Observationally, fat mass was strongly positively related to BMD at all sites, but more weakly at the skull. Instrumental variables (IV) analyses using an allelic score of BMI SNPs suggested that fat mass was causally related to LL-BMD, UL-BMD, SP-BMD and PE-BMD but not SK-BMD. Multivariable MR, Egger regression and IV analyses involving the *ADCY3* variant suggested a positive causal effect of adiposity on all sites except the skull, and that an effect was present even after taking lean mass into account. Finally, IV analyses using BMD allelic scores showed no evidence of reverse causality between BMD and fat mass.

**Conclusions:** Our results suggest that adiposity is causally related to increased BMD at all sites except the skull, perhaps reflecting positive effects of loading on bone formation at weighted but not unweighted sites. In contrast, we found no evidence for BMD causally affecting BMI or measures of adiposity. Our results illustrate how MR can be used profitably to investigate clinical questions relevant to osteoporosis.

Key Messages

- Mendelian randomization approaches suggest that adiposity is causally related to increased bone mineral density of the limbs, pelvis and spine, but not the skull.
- This relationship may reflect positive effects of loading on bone formation at weighted but not unweighted sites.
- No evidence of reverse causality was detected, suggesting that bone mineral density is not causally related to adiposity.
- Mendelian randomization can be used profitably to investigate clinical questions relevant to osteoporosis and bone health.

## Introduction

**Figure 1:**
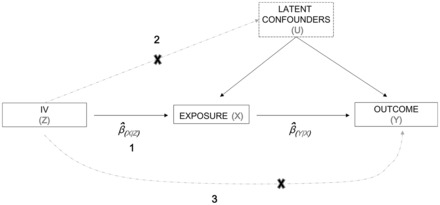
Directed acyclic graph illustrating core instrumental variable assumptions of the Mendelian randomisation approach. The SNP/allelic score used as an instrumental variable (Z) is (1) associated with the exposure of interest (X), (2) independent of unmeasured confounders (U), and (3) independent of the outcome (Y) given the exposure and unmeasured confounding factors. Estimates of the causal effect of the exposure on the outcome can be obtained using a number of estimators including the ratio of the estimated instrumental variable and outcome association to the instrumental variable and exposure association.

Mendelian randomization (MR) is an epidemiological method that uses genetic variants robustly associated with a modifiable exposure or biological intermediate of interest to estimate the causal relationship between these variables and a medically relevant outcome.^1^ The basic principle utilized in MR is that if genetic variants either alter the level of or mirror the biological effects of (i.e. through linkage disequilibrium) a modifiable exposure that itself alters disease risk, then these genetic variants should be related to disease risk to the extent predicted by their influence on exposure to the risk factor. Mendel’s Law of Segregation guarantees that genetic variants are transmitted randomly and independently of potentially confounding environmental factors, and Mendel’s Law of Independent Assortment implies that genetic variants should also segregate independently of other traits provided certain assumptions are met. This randomization achieved through the process of segregation and assortment means that MR studies share many similarities with randomized controlled trials and are often robust to the issues of confounding and reverse causality which plague traditional observational epidemiological studies. The assumptions underlying the MR approach as well as its limitations have been discussed in detail elsewhere^1–4^ (see also Figure 1).

Previously, Timpson *et al.* (2009)^5^ used the Mendelian randomization paradigm to examine a possible causal relationship between adiposity and bone mass using body mass index (BMI)-associated variants in the *FTO* and *MC4R* genes.^6^^,^^7^ The authors found strong association between variants in *FTO* and *MC4R* and BMD, interpreted as a positive causal effect of adiposity on BMD. However, BMI reflects lean as well as fat mass, and it has subsequently become clear that *FTO* and *MC4R* are likewise associated with both fat and lean mass, possibly reflecting relationships with overall body size. Hence pleiotropic effects on lean body mass may have contributed to observed associations between *FTO* and *MC4R* and BMD.

In order to ascertain whether the results of these MR analyses reflect a true causal effect of adiposity on BMD (as opposed to a causal effect of lean mass on BMD induced through the pleiotropic actions of the *FTO* and *MC4R* variants), we examined the relationship between adiposity and BMD at the skull (SK), upper limbs (UL), spine (SP), pelvis (PE) and lower limbs (LL) using 32 BMI-associated SNPs, including a variant near *ADCY3* that was strongly associated with fat mass but not lean mass in our sample. We argue that if adiposity causally increases BMD, we would expect to see a relationship between BMD and SNPs related to fat mass only. Furthermore, if this causal relationship is mediated by loading (i.e. rather than, say, through an endocrine effect), the causal effect estimate should be strongest at the lower limbs and weakest at the skull.

We also utilized two relatively new extensions of the MR method—multivariable MR^8^ and MR Egger regression^9^ which are more robust to violations of the exclusion restriction criterion (i.e. the assumption of no horizontal pleiotropy) than standard MR—to provide further evidence in support of a causal effect of adiposity on BMD. Briefly, multivariable MR uses multiple genetic variants associated with several measured risk factors to simultaneously estimate the causal effect of each of the risk factors on the outcome.^8^^,^^10^ Intuitively, multivariable MR can be thought of as a two-stage procedure where multiple exposures are first regressed on several genetic instruments in a multivariate regression. In the second stage, the outcome of interest is then regressed on the predicted values from the first-stage regression using multivariable regression, analogous to the two-stage least squares procedure utilized in single variable MR. Multivariable MR makes the critical assumption that the relationship between the genetic instruments and the outcome is mediated exclusively by the exposure variables considered in the analysis, which of course may not be the case in reality. In the present study, we used multivariable MR analysis to estimate the causal effect of lean and fat mass on BMD. Provided the above assumptions are satisfied, the results of this analysis should yield estimates of the (direct) causal effect of fat mass on BMD, even if the genetic variants used in this study also pleiotropically affect lean mass.^8^

A new statistical procedure called MR Egger regression was additionally used to exclude the possibility that the results of our MR analyses were contaminated due to violations of the restriction exclusion assumption through horizontal pleiotropy (i.e. adiposity associated variants also directly influence lean mass which then influences BMD). MR Egger regression is more flexible than multivariable MR in that there is no requirement to measure potential pleiotropic pathways directly. Rather, the procedure involves regressing estimates of the instrument-outcome association on estimates of the instrument-exposure association. Provided certain assumptions are met, the slope of the weighted regression line provides an estimate of the causal effect of the exposure on the outcome free from the effects of horizontal pleiotropy. The intercept in the regression is a function of extent of directional pleiotropy in the data aggregated across all the different variants used in the analysis, and statistical tests of the degree to which the intercept differs from zero are akin to testing for the overall presence of directional pleiotropy in the data. However, the validity of MR Egger regression rests on the ‘INSIDE assumption’ (INstrument Strength is Independent of Direct Effect) which states that across all instruments there should be no correlation between the strength with which the instrument proxies the exposure of interest, and its degree of association with the outcome via pathways other than through the exposure. This is a weaker requirement than the exclusion restriction criterion in normal MR which postulates that SNPs may only influence the outcome through the exposure of interest, and so MR Egger regression is likely to be more robust to horizontal pleiotropy than standard MR approaches, although this appears to come at the cost of decreased power to detect a causal effect.^9^ In the context of the present study, provided the underlying assumptions are met, the slope of the MR Egger regression analysis should yield an estimate of the causal effect of adiposity on BMD that is free from any confounding effects due to horizontal pleiotropy (i.e. regardless of whether horizontal pleiotropy is mediated through lean mass or not).

Finally, we investigated whether our sample showed any evidence for reverse causality (i.e. BMD causally influencing BMI/adiposity) by performing bidirectional MR^11^^,^^12^ in which we examined the relationship between SNPs that proxy BMD, and through BMD their possible effect on BMI/adiposity.

## Methods

### Subjects

ALSPAC is a geographically based UK cohort that recruited pregnant women residing in Avon (South West England) with an expected date of delivery between 1 April 1991 and 31 December 1992. A total of 15 247 pregnancies were enrolled, with 14 775 children born.^13^^,^^14^ Of these births, 14 701 children were alive at 12 months. The present study is based on research clinics to which the whole cohort was invited, held when participants were aged a mean of 9.9 years. Ethical approval was obtained from the ALSPAC Law and Ethics committee, and the local research ethics committees. Parental consent and child’s assent were obtained for all measurements made. Please note that the study website contains details of all the data that are available through a fully searchable data dictionary [http://www.bris.ac.uk/alspac/researchers/data-access/data-dictionary/].

### Total-body dual-energy X-ray absorptiometry and anthropometric measures

Total-body dual-energy X-ray absorptiometry (TB-DXA) scans were performed on all participants at the age 9.9 years clinic, using a Lunar Prodigy scanner (Lunar Radiation Corp, Madison, WI) with paediatric scanning software (GE Healthcare Bio-Sciences Corp., Piscataway, NJ). Dual-energy X-ray absorptiometry (DXA) measures of BMD were derived for the following regions of interest: total body less head (TBLH), skull (SK), upper limb (UL), lower limb (LL), spine (SP) and pelvis (PE). All DXA scans were subsequently reviewed by a trained researcher and re-analysed as necessary, to ensure that borders between adjacent regions of interest (ROIs) were placed correctly by the automated software. The coefficient of variation for TBLH-BMD measures was 0.8%, based on the analysis of 122 children who had two scans performed on the same day. Data on lean mass and fat mass were also obtained from the above-mentioned scans. Height was measured to the nearest 0.1 cm using a Harpenden stadiometer (Holtain Ltd, Crymych, UK) and weight was measured to the nearest 50 g using Tanita weighing scales (Tanita UK Ltd, Uxbridge). BMI was derived as a ratio of body mass (kg) to height squared (m^2^).

### Genetic data

A total of 9912 subjects were genotyped using the Illumina HumanHap550 quad genome-wide SNP genotyping platform (Illumina Inc., San Diego, CA, USA) by Logistics and Genotyping Facilities at the Wellcome Trust Sanger Institute and Laboratory Corporation of America (LabCorp Holdings, Burlington, NC, USA) using support from 23 and Me. PLINK software (v1.07) was used to carry out quality control measures.^15^ Individuals were excluded from further analysis on the basis of having incorrect gender assignments, minimal or excessive heterozygosity (< 0.320 and > 0.345 for the Sanger data and < 0.310 and > 0.330 for the LabCorp data), disproportionate levels of individual missingness (> 3%), evidence of cryptic relatedness (> 10% Identity by descent (IBD)) and being of non-European ancestry (as detected by a multidimensional scaling analysis seeded with HapMap 2 individuals). EIGENSTRAT analysis^16^ revealed no additional obvious population stratification and genome-wide analyses with other phenotypes in the same cohort indicate a low lambda. SNPs with a minor allele frequency of < 1% and call rate of < 95% were removed. Furthermore, only SNPs that passed an exact test of Hardy–Weinberg equilibrium (*P* > 5 × 10^−7^) were considered for analysis. After quality control, 8365 unrelated individuals who were genotyped at 500 527 SNPs were available for analysis. Known autosomal variants were imputed with Markov Chain Haplotyping software (MACH 1.0.16)^17^^,^^18^ using Centre d'Etude du Polymorphisme Humain (CEPH) individuals from phase II of the HapMap project (hg18) as a reference set (release 22).^19^

### Cross-sectional analyses

All statistical analyses were performed using datasets restricted to children with complete data across all genotypic and phenotypic variables (5221 participants). Observational associations between BMD across five body sites (SK, UL, SP, PE and LL) and BMI, total body fat mass and total body lean mass were estimated using ordinary least squares linear regression while controlling for age and sex.

SNPs robustly related to BMI on the basis of a previous genome-wide association study,^20^ as well as those related to BMD^21^ were extracted from the complete set of ALSPAC genome-wide imputed genotypes in order to proxy BMI, hip-BMD and spine-BMD (see Supplementary Tables 1, 2 and 3 for a list of SNPs used to proxy each trait, available as Supplementary data at *IJE* online). Since the majority of SNPs only explained small amounts of variance in our variables, we also constructed allelic scores of SNPs in order to better proxy our exposures of interest. Unweighted allelic scores were calculated as a simple count of the number of trait increasing alleles. For bidirectional IV analyses, two allele scores were generated—one consisting of SNPs associated with bone mineral density at the femoral neck of the hip (FN-BMD) and one consisting of SNPs associated with bone mineral density at the lumbar spine (LS-BMD) using genome-wide significant SNPs from a study by Estrada and colleagues^21^—see Supplementary Tables 2 and 3.

### Instrumental variables regression

In order to generate estimates of the causal effect of BMI/adiposity on BMD at the various body sites, instrumental variables analyses were performed using two-stage least squares. Analyses were performed both for single variants and for allelic scores. Within these models, sex and age were included as covariates in order to generate estimates from the IV analyses that were comparable to those from the observational regressions. IV estimates were then contrasted to those from ordinary linear regression using the Durbin form of the Durbin-Wu-Hausman statistic. We also examined instrument strength by deriving F-statistics from the first stage regressions (i.e. *F_first_*). As a rule of thumb, F-statistics greater than 10 are often taken to indicate adequate strength to mitigate against any bias of the causal IV estimate.^22^ To investigate the possibility of reverse causation, we performed bidirectional MR^12^ to examine the causal effect of BMD (measured at the skull, spine, pelvis, upper and lower limbs) on BMI and total body fat mass. For these analyses, BMI and fat mass were log transformed to normality. Standard MR analyses assume that genetic instruments only influence the outcome (i.e. BMD) through the exposure of interest (i.e. fat mass). However, BMI-associated SNPs may influence BMD through pathways other than adiposity, including through effects on lean mass. We therefore tested the robustness of our results by utilizing two relatively new extensions of the MR method, multivariable MR^8^^,^^10^ and MR Egger regression^9^ which, provided certain assumptions are met, help to control for biases brought about through horizontal pleiotropy.

### Multivariable Mendelian randomization

We used multivariable MR to estimate the causal effect of fat mass and lean mass on BMD using a two-stage least squares approach as implemented in R using the Applied Econometrics with R (AER) software package. In the first stage of the analysis, fat mass and lean mass were regressed on the genetic instruments using multivariate regression. In the second stage of the analysis, BMD was regressed on the predicted values from the first stage using multivariable regression. Models were fitted for SK, UL, LL, SP and PE separately. We note that the multivariable MR method does not require every genetic instrument to be related to every risk factor, merely that the instruments are not related to the outcome (i.e. BMD) through paths other than through the risk factors of interest (i.e. lean mass and fat mass). However, multivariable MR is susceptible to weak instrument bias. In order to minimize this possibility, we only used seven of the most strongly related variants from our first-stage univariate analyses (*FTO, ADCY3, MC4R, BCDIN3D, SEC16B, TMEM18* and *TNNI3K*) that showed robust associations with either fat mass and/or lean mass (i.e. *F_first_* > 10, see Supplementary Table 1).

### Mendelian randomization Egger regression

The other strategy we employed to evaluate the robustness of our results was MR Egger regression.^9^ In this procedure, we first fitted univariate regressions of BMD on each of the 32 BMI-related genetic instruments and recorded the value of the slope coefficient and standard error for each variant. We then did the same for univariate regressions of BMI/fat mass on each of the 32 instruments. Finally, we performed a weighted linear regression of the estimated slopes from the first set of regressions (i.e. BMD on SNPs) on the estimated slopes from the second set of regressions (i.e. BMI fat mass on SNPs). The weights for this analysis were the standard errors of the regression of BMD on the relevant genetic variant. We used all 32 BMI-related SNPs in this analysis, and examined the relationship between BMI and fat mass with SK-, UL-, LL-, SP- and PE-BMD. The presence of directional pleiotropy was assessed by evaluating the significance of the intercept term in the above regression and visually inspecting funnel plots. All statistical analyses were performed with *R* version 3.02^23^ using the following software packages: AER, reshape and ggplot2.

## Results

### Observational relationships of BMI, and fat mass, on BMD

A total of 5221 children (2561 males, 2660 females) were identified in ALSPAC who had DXA, anthropometric and genetic data recorded at mean age 9.9 years. (Note: there were 4223 children (2116 males and 2107 females) in the case of SP-BMD only.). Lean mass was greater in males, whereas fat mass was greater in female participants (Table 1). BMI, height and weight were similar in both genders. BMD at all regions of interest was similar across the sexes. Observationally, BMI, fat mass and lean mass were positively associated with BMD measured across all skeletal sites. Standardized regression coefficients (with their 95% confidence intervals) for all three ‘exposures’ were consistently lower at the skull compared with the other sites after adjustment for age and sex (Table 2).

**Table 1.** The descriptive statistics of dual-energy X-ray absorptiometry and anthropometric measures of participants who attended the age 9 focus clinic of the Avon Longitudinal Study of Parents and Children

		**Male (*N* = 2561)**					**Female (*N* = 2660)**				
**Measure**	**Unit**	**Mean**	**SD**	**P25**	**Media*n***	**P75**	**Mean**	**SD**	**P25**	**Media*n***	**P75**
Age	Years	9.94	0.32	9.73	9.87	10.06	9.94	0.32	9.73	9.87	10.08
Height	cm	139.94	6.17	135.80	139.90	144.10	139.40	6.52	134.90	139.10	143.60
Weight	kg	34.47	7.20	29.40	32.80	38.00	34.98	7.59	29.60	33.40	39.20
BMI	kg/m^2^	17.49	2.77	15.60	16.72	18.78	17.88	2.94	15.75	17.24	19.52
Fat mass	kg	7.41	4.92	3.93	5.71	9.58	9.66	5.05	5.92	8.41	12.22
Lean mass	kg	25.51	2.94	23.48	25.36	27.41	23.62	3.14	21.51	23.30	25.31
SK-BMD	g/cm^2^	1.59	0.14	1.50	1.59	1.68	1.56	0.14	1.46	1.55	1.65
UL-BMD	g/cm^2^	0.66	0.04	0.63	0.66	0.69	0.65	0.04	0.62	0.65	0.67
LL-BMD	g/cm^2^	0.90	0.08	0.85	0.90	0.96	0.89	0.08	0.84	0.89	0.95
SP-BMD[Fn dyw079-TF3]	g/cm^2^	0.77	0.08	0.72	0.76	0.82	0.78	0.08	0.71	0.77	0.83
PE-BMD	g/cm^2^	0.83	0.07	0.78	0.83	0.88	0.83	0.07	0.78	0.83	0.88

Parameters presented as mean, standard deviation (SD), median, 25th (p25) and 75th (p75) centiles.

Age, age at DXA scan; fat mass, DXA-derived total body fat mass; lean mass, DXA-derived total body lean mass; BMD, DXA-derived bone mineral density.

^a^4223 subjects were available for analyses involving the spine (2116 males and 2107 females).

**Table 2.** Summary statistics describing observational (OBS) and causal relationships between BMI/fat mass and BMD measured at several skeletal sites

		**BMI**					**Fat mass**				
**Trait**	**Method**	$\hat{\beta }$	**CI-L**	**CI-U**	***P***	***P*_WH_**	$\hat{\beta }$	**CI-L**	**CI-U**	***P***	***P*_WH_**
SK-BMD	OBS	0.19	0.17	0.22	< 0.001	–	0.19	0.16	0.22	< 0.001	–
	TSLS	−0.02	−0.20	0.15	0.78	0.01	−0.03	−0.22	0.16	0.78	0.02
	MR-E	0.14	−0.11	0.40	0.29	–	−0.03	−0.29	0.24	0.84	–
											
UL-BMD	OBS	0.49	0.47	0.51	< 0.001	–	0.46	0.44	0.49	< 0.001	–
	TSLS	0.46	0.31	0.61	< 0.001	0.69	0.51	0.34	0.67	< 0.001	0.60
	MR-E	0.51	0.33	0.69	< 0.001	–	0.57	0.37	0.77	< 0.001	–
											
LL-BMD	OBS	0.59	0.57	0.61	< 0.001	–	0.59	0.56	0.61	< 0.001	–
	TSLS	0.55	0.41	0.68	< 0.001	0.51	0.60	0.45	0.75	< 0.001	0.87
	MR-E	0.62	0.44	0.80	< 0.001	–	0.73	0.55	0.91	< 0.001	–
											
SP-BMD^[Fn dyw079-TF7]^	OBS	0.54	0.52	0.57	< 0.001	–	0.53	0.50	0.56	< 0.001	–
	TSLS	0.48	0.33	0.63	< 0.001	0.42	0.52	0.35	0.69	< 0.001	0.94
	MR-E	0.61	0.40	0.83	< 0.001	–	0.49	0.29	0.70	< 0.001	–
											
PE-BMD	OBS	0.47	0.44	0.49	< 0.001	–	0.45	0.43	0.48	< 0.001	–
	TSLS	0.39	0.24	0.54	< 0.001	0.31	0.43	0.26	0.60	< 0.001	0.79
	MR-E	0.49	0.34	0.64	< 0.001	–	0.47	0.31	0.62	< 0.001	–

Separate methods were used to investigate causal relationships, namely: two-stage least squares regression (TSLS) and MR-Egger regression (MR-E). Effect sizes ($\hat{\beta }$) expressed as SD change in outcome per SD change in exposure with the upper (CI-U) and lower (CI-L) 95% confidence estimate of$\;\hat{\beta }$.

Observationally, lean mass was positively associated with BMD measured at the SK [$\hat{\beta }$= 0.25 (0.22 - 0.28)], UL [$\hat{\beta }$= 0.59 (0.56 - 0.61)], LL [$\hat{\beta }$= 0.72 (0.70 - 0.74)], SP [$\hat{\beta }$= 0.60 (0.58 - 0.63)] and PE [$\hat{\beta }$= 0.66(0.64 - 0.68)].

*P*, strength of evidence against the null hypothesis of no association between the outcome (BMD) and exposure variables (BMI/fat mass); *P*_WH_, the strength of evidence against the null hypothesis of no endogeneity (i.e. no difference in effect size of the observational and the causal effect of BMI/fat mass on BMD); fat mass, DXA-derived total body fat mass; BMD, DXA-derived bone mineral density; SK, skull; UL, upper limbs; LL, lower limbs; SP, spine; PE, pelvis.

^a^4223 subjects were available for analyses involving the spine (2116 males and 2107 females).

Supplementary Table 1 presents first-stage regression results for the BMI-associated SNPs with BMI and fat mass in 5221 children, as well as the association with lean mass for comparison. The majority of SNPs showed at least nominal association (*P* ≤ 0.05) with BMI and fat mass in the expected direction of association. However for most SNPs the effect was not strong, suggesting that many of these variants might suffer from appreciable weak instrument bias in our sample (i.e. *F_first_* < 10). Notable exceptions included variants at the *FTO, ADCY3, MC4R, BCDIN3D, SEC16B, TMEM18* and *TNNI3K* loci which were all strongly associated with BMI. First-stage regression results were similar for fat mass also, suggesting that many of these SNPs adequately proxy adiposity. However, the majority of SNPs also showed at least nominal association with lean mass, including the variants in *FTO* and *MC4R* which have been used in previous MR studies to proxy adiposity specifically.^5^ Interestingly, the SNP at *ADCY3* was the only variant that showed strong association with BMI and fat mass (*F_first_* = 26), but no evidence of association with lean mass in our sample. Allelic scores comprising all 32 variants were strongly related to BMI (*F_first_* = 134) and fat mass (*F_first_* = 120), but also showed considerable association with lean mass [(*F_first_* = 46), Supplementary Table 1].

### Instrumental variable estimates of causal effects of BMI, and fat mass, on BMD

Supplementary Tables 4 and 5 (available as Supplementary data at *IJE* online) present results for the IV regressions using single instruments and allele scores for BMI and adiposity. Each IV regression coefficient estimate represents the causal change in standard deviations of BMD per standard deviation change in BMI/fat mass. Variants most strongly related to BMI/adiposity produced strong positive estimates for the causal effect of BMI and adiposity on UL-, SP-, PE- and LL-BMD. However, their association with SK-BMD was much more equivocal, with some variants producing positive causal estimates (e.g. *MC4R*, *TMEM18*), whereas others displayed neutral or even negative estimates of the causal effect (e.g. *SLC39A8* and *BCD1N3D*). Interestingly, IV analyses involving the fat mass-specific *ADCY3* variant showed strong evidence of a positive causal effect of fat mass on BMD at the lower-limbs [${\hat{\beta }}_{IV}$ = 0.44 (0.11 - 0.77)], but not the skull, although the confidence intervals were wide [${\hat{\beta }}_{IV}$ = –0.01 (–0.41 - 0.39)]. The point estimate of the causal relationship between fat mass and UL-, SP- and PE-BMD using the *ADCY3* variant was intermediate between these two estimates, but the confidence intervals overlapped zero [UL-BMD: ${\hat{\beta }}_{IV}$ = 0.23 (–0.13 - 0.59); SP-BMD: ${\hat{\beta }}_{IV}$ = 0.25 (–0.18 - 0.68); PE-BMD: ${\hat{\beta }}_{IV}$ = 0.18 (–0.19 - 0.55)].

As noted in the previous section, many of the individual variants were not strongly related to BMI or fat mass in our sample (*F_first_* < 10), and so any estimates of the causal effect derived from these analyses are likely to be biased towards the observational association through the influence of weak instrument bias. We therefore combined all the BMI-associated variants into a single unweighted allelic score. The results of the IV regressions using the allele score as an instrument suggested that BMI and adiposity were causally associated with BMD measured at the lower and upper limbs, spine and pelvis, but not at the skull (see Table 2). The magnitude of the estimated causal effect was larger at the lower limbs compared with other regions.

### Multivariable instrumental variables analysis

Multivariable IV analysis provided additional evidence of a causal effect of fat mass on lower limb BMD, independent of the effects of lean mass [fat mass: ${\hat{\beta }}_{IV}$ = 0.40 (0.18 - 0.62); lean mass: ${\hat{\beta }}_{IV}$ = 0.54 (0.19 - 0.89)]. A similar trend was observed for the upper limbs [fat mass: ${\hat{\beta }}_{IV}\;$= 0.21 (−0.06 - 0.47); lean mass: ${\hat{\beta }}_{IV}$ = 0.68 (0.26 - 1.11)], spine [fat mass: ${\hat{\beta }}_{IV}\;$= 0.31 (0.02 - 0.60); lean mass: ${\hat{\beta }}_{IV}$ = 0.44 (−0.02 - 0.90)], and pelvis [fat mass: ${\hat{\beta }}_{IV}\;$= 0.24 (0.00 - 0.50); lean mass: ${\hat{\beta }}_{IV}$ = 0.50 (0.11 - 0.90)]. However, in many cases the 95% confidence intervals for the causal estimate for fat mass just overlapped zero. There was no evidence for a causal effect of fat mass at the skull [${\hat{\beta }}_{IV}$ = -0.08 (−0.40 - 0.24), *P* = 0.61], although there was some evidence for a causal effect of lean mass at this site [${\hat{\beta }}_{IV}$ = 0.45 (−0.05 - 0.96)].

### MR Egger regression analysis

**Figure 2:**
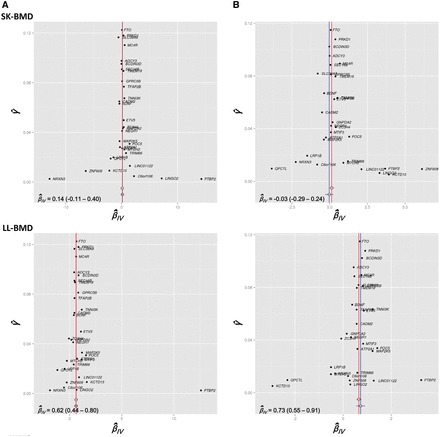
Funnel plots displaying the strength of association between each of 32 SNPs ($\hat{\gamma }$) with BMI (Panel A) and Fat mass (Panel B) plotted against the causal estimate ($\hat{\beta }$_IV_) of each SNP on BMD measured at the skull (SK) and lower-limbs (LL). The inverse-variance weighted and MR Egger causal effect estimates are represented by a red and blue line respectively.

**Figure 3:**
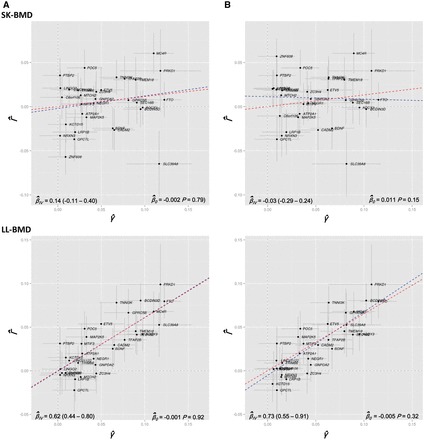
Scatter plots displaying estimates of the association between each SNP and the relevant BMD outcome ($\hat{\Gamma }$) against effect estimates of each SNP with the relevant exposure [i.e BMI (panel A) and Fat mass (panel B)]. The slope of the blue line through the plot represents the MR Egger regression estimate ($\hat{\beta }$_IV_) of the causal effect of the exposure on the outcome. The y-intercept of the blue regression line denotes the estimate of the degree of directional pleiotropy in the dataset ($\hat{\beta }$_0_). The inverse-variance weighted causal effect estimate is represented by the slope of the red line.

The funnel plots in Figure 2 display MAF-corrected genetic associations between each of the individual SNPs and BMI (panel A) / adiposity (panel B) plotted against their causal effect estimates. Visual inspection of the plots provided little evidence for the existence of directional pleiotropy across the different skeletal sites. This interpretation was corroborated by formal statistical tests of the intercept from the MR Egger regression analyses (Supplementary Table 6, available as Supplementary data at *IJE* online). Figure 3 illustrates the associations between the BMI related variants and BMI (panel A) / adiposity (panel B) with BMD at the different skeletal sites in the form of scatter diagrams. The slope of the line through the plot is the MR Egger regression estimate of the causal effect using all variants as instrumental variables. Egger regression produced strong estimates of the causal effect of BMI and fat mass on LL-BMD [BMI: ${\hat{\beta }}_{IV}$ = 0.62 (0.44 – 0.80); fat mass: ${\hat{\beta }}_{IV}$ = 0.73 (0.55 – 0.91), Table 2, Figure 2 and 3], UL-BMD [BMI: ${\hat{\beta }}_{IV}$ = 0.51 (0.33 – 0.69); fat mass: ${\hat{\beta }}_{IV}$ = 0.57 (0.37 – 0.77)], SP-BMD [BMI: ${\hat{\beta }}_{IV}$ = 0.61 (0.40 – 0.83); fat mass: ${\hat{\beta }}_{IV}$ = 0.49 (0.29 – 0.70)] and PE-BMD [BMI: ${\hat{\beta }}_{IV}$ = 0.49 (0.34 – 0.64); fat mass: ${\hat{\beta }}_{IV}$ = 0.47 (0.31 – 0.62)] (Table 2; Supplementary Figures 1 and 2, available as Supplementary data at *IJE* online). In contrast, whilst there was some evidence for a causal relationship between BMI and SK-BMD [${\hat{\beta }}_{IV}$ = 0.14 (−0.11 - 0.40), although the 95% confidence intervals overlapped zero], there was little evidence for a causal effect of fat mass on SK-BMD [${\hat{\beta }}_{IV}$ = -0.03 (−0.29 - 0.24), *P* = 0.84] (Table 2, Figures 2 and 3).

## Bidirectional MR analyses

Finally we investigated whether there was any evidence for BMD having a causal effect on BMI/adiposity. Supplementary Tables 2 and 3 present the first-stage regression results for FN- and LS-BMD associated SNPs with BMD measures at the skull, upper and lower limbs, spine and pelvis. Many FN-BMD associated SNPs showed at least nominal association (*P* ≤ 0.05) with BMD measured across the different sites, consistent with previously published work.^24^ However for most SNPs, the effect was not strong, suggesting that many of these variants might suffer from weak instrument bias in our sample if used singly (i.e. *F_first_* < 10). A similar trend was observed for LS-BMD associated variants.

Subsequently, we combined FN- and LS-BMD associated variants into two separate unweighted allelic scores and observed that both were robustly associated with BMD measured at the skull (*F_first_*_-_*_FN_* = 123 and *F_first-LS_* = 176), upper limbs (*F_first_*_-_*_FN_* = 81 and *F_first-LS_* = 86), lower limbs (*F_first_*_-_*_FN_* = 79 and *F_first-LS_* = 51), spine (*F_first_*_-_*_FN_* = 73 and *F_first-LS_* = 79) and pelvis (*F_first_*_-_*_FN_* = 93 and *F_first-LS_* = 107) (see Supplementary Tables 2 and 3). Reciprocal IV analyses using BMD allelic scores showed no evidence of reverse causality occurring between BMD at any of the skeletal sites and BMI or fat mass (see Supplementary Tables 7–16, available as Supplementary data at *IJE* online).

## Discussion

Our results using a variety of MR techniques involving single and multiple genetic instruments suggest a strong causal effect of adiposity on bone mineral density at the lower limbs, upper limbs, spine and pelvis. A causal effect was apparent regardless of analysis (i.e. single variant MR, using an allelic score of 32 BMI-associated variants, using MR Egger regression or multivariate MR) and was present even for a variant at the *ADCY3* locus that showed a relationship with fat mass but not lean mass in our sample. In contrast, MR analyses failed to show convincing evidence of a relationship between adiposity-associated SNPs and skull BMD, the only exception being multivariable MR analysis which suggested that any causal effect on BMD was likely to be mediated through an effect of lean mass rather than adiposity. We also found no evidence for the reciprocal relationship—that is, BMD having a causal effect on BMI/adiposity.

One of the key assumptions underlying the MR approach is that the SNPs used as genetic instrumental variables are only related to the outcome of interest through the exposure variable under study. Within the context of the present study, this means that standard MR assumes no pleiotropic pathways from adiposity-related SNPs to BMD that pass through intermediates other than adiposity (Figure 1). We have shown that this assumption is unlikely to be fulfilled for the majority of BMI-associated variants from the Speliotes *et al.* (2010) paper, since many of these SNPs also appear to show varying degrees of association with lean muscle mass as well as adiposity. This includes two BMI-related variants, that were used in a previous MR study of the same phenotypes, which exhibit non-trivial associations with lean mass in our sample.^5^

A notable exception appears to be a variant near *ADCY3* which was very strongly associated with fat mass/BMI in our sample, but showed no evidence of association with lean mass. The existence of this SNP provides an opportunity to test the hypothesis that adiposity causally affects BMD independently of lean mass. Our results suggest that this was indeed the case, and the fact that this effect was present at the lower limbs and other body sites subject to loading (i.e. the upper limbs, spine and pelvis), but not the skull, suggests that a causal effect of fat mass on BMD was likely to be mediated through an effect of loading rather than an endocrine mechanism. Additionally, whereas the bones of the arms and legs differ from the skull in terms of their type, composition and ossification processes, the skull and pelvis share many similarities in these regards. Given our analyses imply an effect of BMI/adiposity on pelvic but not skull BMD, the most likely explanation for a causal relationship between BMI/adiposity and BMD is loading.

There are several qualifications to this interpretation. First, although the point estimate for the causal effect of adiposity on skull BMD using this variant was close to zero, the confidence intervals on the causal estimate were wide. Thus our findings would benefit from replication in a larger sample of individuals as well as utilizing SNPs that are specific (and strongly associated with) lean or fat mass. Unfortunately, the common variants related to adiposity identified in more recent genome-wide association studies (GWAS) are even weaker than the ones used in this study,^25^ and there are currently no known variants specifically associated with lean mass, although there are GWAS under way to identify such associations.

In addition we cannot rule out the possibility that the *ADCY3* variant may exhibit a small positive association with lean mass in larger samples (i.e. the confidence intervals in ALSPAC do not exclude a small effect on lean mass), or indeed that the SNP exerts effects on BMD that are mediated via pathways independent of adiposity.^26^ For example, the *ADCY3* variant has previously shown a relationship with adult height,^27^ although the direction of association was in the opposite direction to its association with BMI. In ALSPAC, the variant is strongly related to total body fat mass but only shows nominal evidence of association with height, as previously reported.^28^

Given that our results for single adiposity-related variants, the single SNP *ADCY3* and the allelic score of adiposity-related SNPs are potentially open to confounding influences, we employed two statistical procedures to mitigate concerns due to horizontal pleiotropy—MR Egger regression^9^ and multivariable MR.^8^ Both of these procedures produced results very similar to our other analyses. The validity of the MR Egger approach depends on the degree to which the INSIDE assumption is satisfied—that is, it assumes that there is no correlation between the strength of instrument and the strength of association with the outcome via pathways other than through the exposure. Simulations in Bowden suggest that violation of the INSIDE assumption will result in biased estimates of the true causal effect, depending on a number of factors including the degree of horizontal pleiotropy present in the data. Variants that show the strongest association with BMI/fat mass also tend to show association with lean mass (although this is not always true, as shown by the *ADCY3* variant).

It is also unclear the extent to which weak instrument bias has effects on MR Egger regression. Many of the variants used in our analysis are likely to suffer from this bias. We have performed limited simulations suggesting that the MR Egger method is susceptible to weak instrument bias, with the exact effects depending on the number of weak instruments used, whether two-sample or single-sample MR is employed and the nature of pleiotropy in the dataset (data not shown). Specifically, the bias appears to increase with increasing numbers of weakly associated variants and is a key reason why we did not perform these analyses using a far larger number of very weak instruments identified in a more recent genome-wide scan of BMI.^25^

Finally, bidirectional MR analyses produced no evidence for the reverse relationship (i.e. BMD causing BMI/adiposity). Genetic instruments for these analyses were selected from a large genome-wide association study of BMD in which weight was included as a covariate.^21^ This has the advantage of selecting variants whose association with BMD is not mediated through BMI/weight (i.e. the variant’s primary association is with BMD). There is a remote possibility that some of these variants are weight variants and not at all associated with BMD, but rather that the association is induced by a collider effect induced by conditioning on weight.^29^ However, we consider this possibility to be unlikely as there is little evidence that these variants are associated with BMI, as noted in very large studies of the trait.^20^^,^^25^

**Figure 4:**
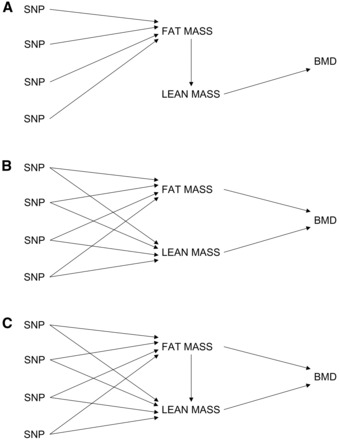
Directed acyclic graphs illustrating three scenarios that potentially account for the causal relationship between adiposity and bone mineral density (BMD), in addition to the shared associations with lean mass. Panel A depicts a scenario in which the causal influence of adiposity on BMD is mediated by lean mass. Panel B depicts a scenario in which SNPs affecting adiposity also directly influence lean mass via horizontal pleiotropy, and both fat mass and lean mass have direct causal effects on BMD. Panel C depicts a combination of the scenarios illustrated by panel A and B.

In aggregate, the results of our MR analyses suggest that adiposity causally affects BMD. What is unclear, however, is whether the causal effect of adiposity on BMD is mediated by lean mass (see Figure 4A), or whether SNPs that influence adiposity influence fat mass via horizontal pleiotropy (see Figure 4B), or perhaps a combination of these mechanisms (Figure 4C). In favour of the first possibility is the observation that many SNPs that affect fat mass, also show evidence of association with lean mass (under this model, given a large enough sample size we would expect that all SNPs associated with adiposity show association with lean mass). Such a relationship is physiologically plausible too. since increased fat mass may promote increased muscle mass in order to shift increased weight, which would then causally increase BMD.

In contrast, our data provide two good reasons to suggest that adiposity has, at least in part, a direct effect on BMD that is not mediated by muscle. First, the *ADCY3* variant is strongly related to adiposity in our sample, but shows little evidence of association with lean mass. As intimated, if lean mass mediated the relationship between adiposity and BMD we would expect that all variants related to fat mass should show some evidence of association with lean mass (although we acknowledge that the absence of association with lean mass could simply reflect sampling variation in our dataset). Also relevant are the results of the multivariable MR analyses. Even though the multivariable MR model assumes a causal relationship like that illustrated in Figure 4B, Burgess *et al.* (2015)^8^ have shown that in the case of a mediated relationship like in Figure 4A or 4C, multivariable MR analysis produces estimates of the direct effect of the exposure variables on the outcome. In the context of our analyses, this means that multivariable MR produces estimates of the direct effect of adiposity on BMD, as opposed to the total effect of adiposity on BMD (which would also include effects mediated by lean mass). Thus, the fact that the multivariable MR analysis produced significant estimates for a causal effect of fat mass on BMD suggests that adiposity has at least some direct causal effect on BMD. We stress that our conclusions in this regard are not definitive, and indeed our dataset is limited in terms of the information it can provide regarding the nature of mediation. Should genetic instruments that exclusively proxy lean mass become available in the future, then two-step and network MR approaches may be able to shed light on these competing hypotheses.^30^^,^^31^ In summary, we have used a range of different MR procedures in this work, each with its own strengths and limitations, to try to obtain a clearer picture of the nature of the relationship between BMI/adiposity and BMD. The results of these analyses consistently suggest that adiposity is causally related to increased BMD at all body sites except the skull, perhaps reflecting positive effects of loading on bone formation at weighted but not unweighted sites. In contrast, we found no evidence for the reverse relationship, i.e. BMD causally affecting BMI or measures of adiposity. Our results illustrate how MR can be used profitably to investigate clinical questions relevant to osteoporosis and bone health.

## Funding

This work is supported by a Medical Research Council programme grant (MC_UU_12013/4 to D.M.E). The UK Medical Research Council and the Wellcome Trust (Grant refs: 092731 and 102215/2/13/2) and the University of Bristol provide core support for ALSPAC. D.M.E is supported by an Australian Research Council Future Fellowship (FT130101709). GWAS data were generated by Sample Logistics and Genotyping Facilities at the Wellcome Trust Sanger Institute and LabCorp (Laboratory Corporation of America) using support from 23andMe.

## Supplementary Material

Supplementary Data
