# Supplementary material for: Using Mendelian randomization to investigate a possible causal relationship between adiposity and increased bone mineral density at different skeletal sites in children
Source: Int J Epidemiol. 2016 May 22;45(5):1560–72. doi: 10.1093/ije/dyw079 (PMC5100609; doi:10.1093/ije/dyw079)
Supplement: Supplementary Data [file dyw079_supplementary_data.zip › ije-2015-12-1630-File010.docx]

**SUPPLEMENTARY FIGURES LEGENDS**

**Supplementary Figure 1:** Funnel plots displaying the strength of association between each of 32 SNPs ($\hat{\gamma}$) with BMI (Panel A) and Fat mass (Panel B) plotted against the causal estimate ($\hat{\beta}_{IV}$) of each SNP on BMD measured at the upper-limb (UL), spine (SP) and pelvis (PE). The inverse-variance weighted and MR Egger causal effect estimates are represented by a red and blue line respectively.

**Supplementary Figure 2:** Scatter plots displaying estimates of the association between each SNP and the relevant BMD outcome $(\hat{\Gamma})$ against effect estimates of each SNP with the relevant exposure [i.e BMI (panel A) and Fat mass (panel B)]. The slope of the blue line through the plot represents the MR Egger regression estimate ($\hat{\beta}_{IV}$) of the causal effect of the exposure on the outcome. The y-intercept of the blue regression line denotes the estimate of the degree of directional pleiotropy in the dataset ($\hat{\beta}_{0}$). The inverse-variance weighted causal effect estimate is represented by the slope of the red line.
