# Supplementary figures and images for: Using Mendelian randomization to investigate a possible causal relationship between adiposity and increased bone mineral density at different skeletal sites in children
Source: Int J Epidemiol. 2016 May 22;45(5):1560–72. doi: 10.1093/ije/dyw079 (PMC5100609; doi:10.1093/ije/dyw079)

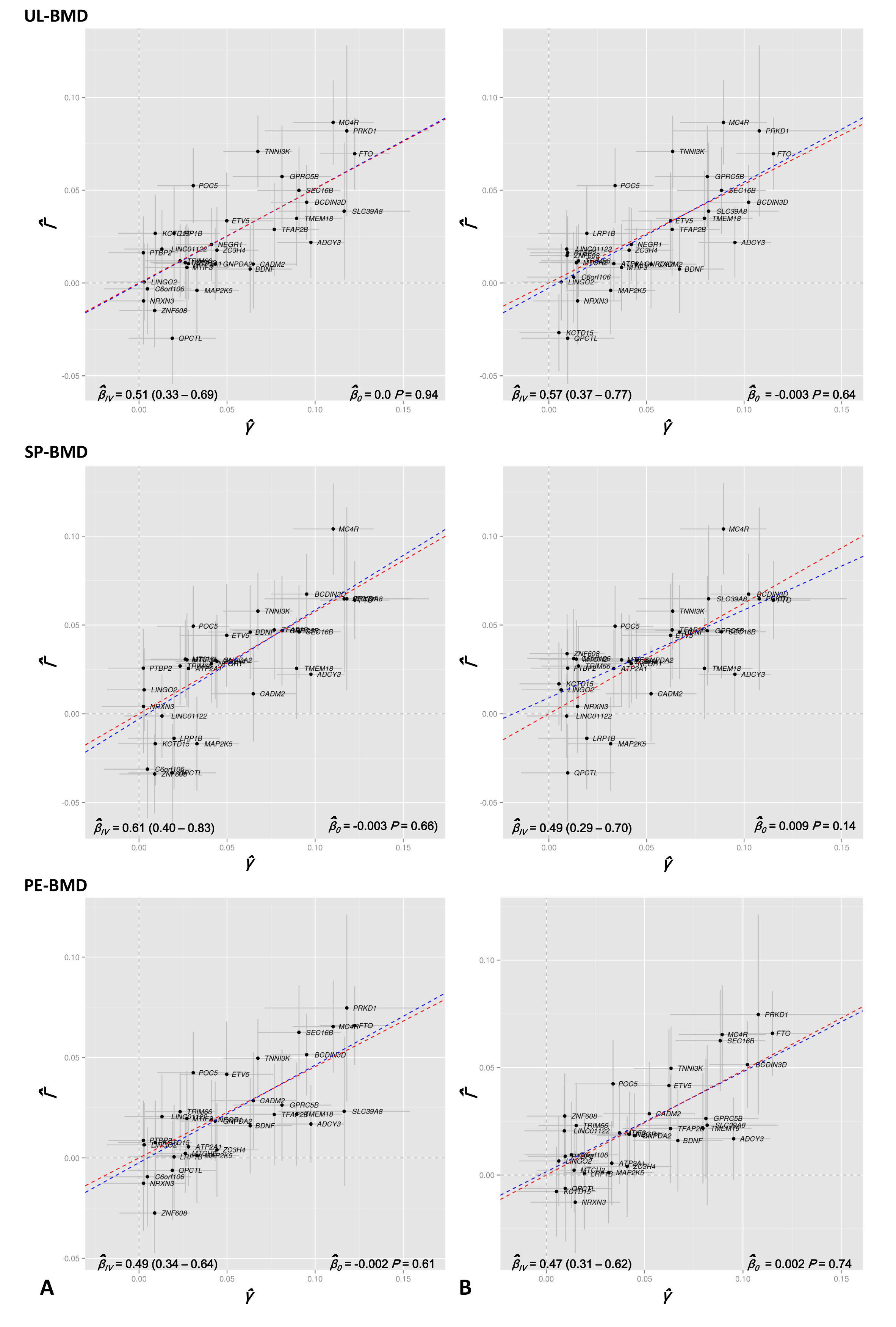

Supplement: Supplementary Data [file dyw079_supplementary_data.zip › ije-2015-12-1630-File009.tif]

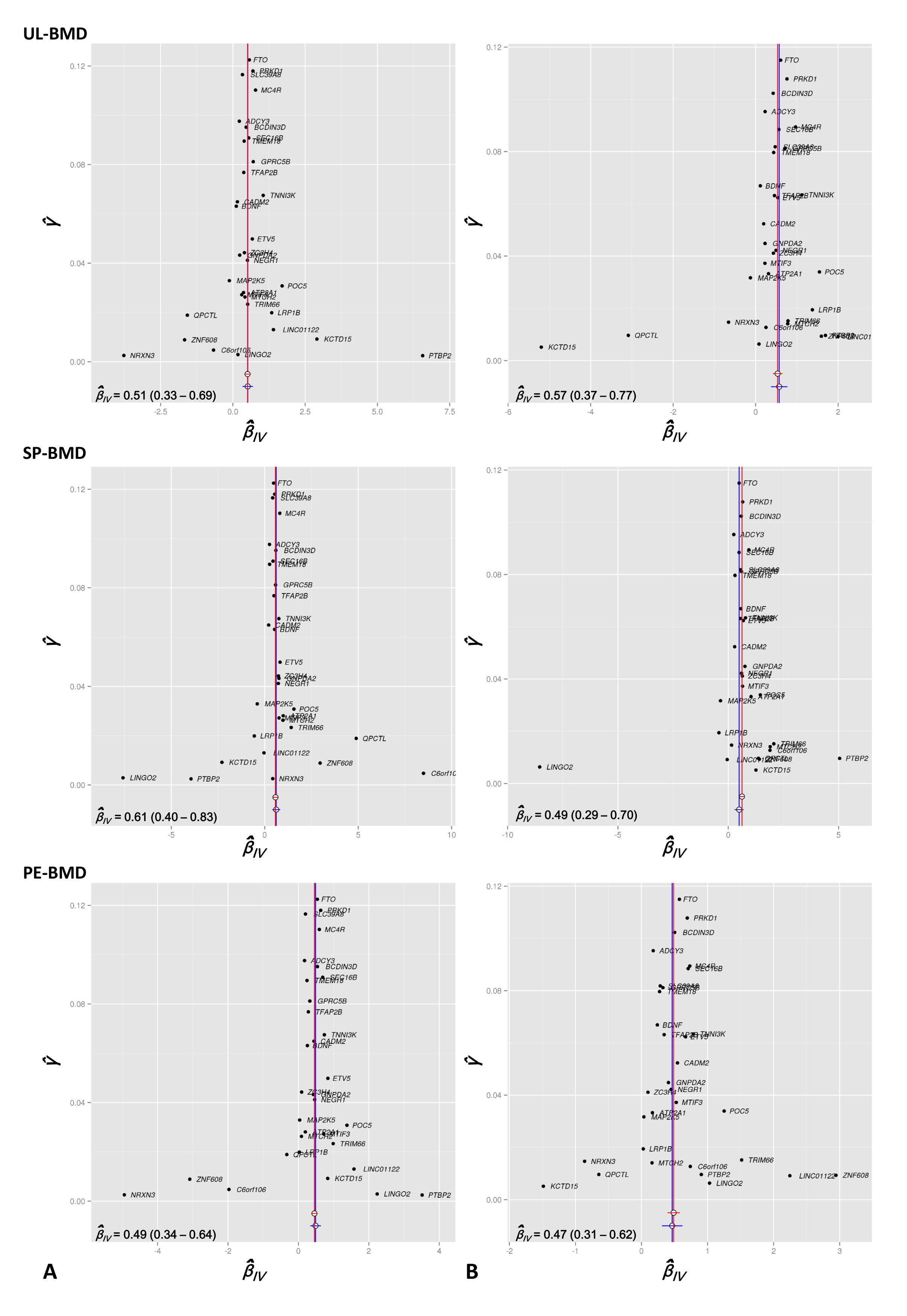

Supplement: Supplementary Data [file dyw079_supplementary_data.zip › ije-2015-12-1630-File008.tif]
